# CT Assessment of Aortopulmonary Septal Defect: How to Approach It?

**DOI:** 10.3390/jcm13123513

**Published:** 2024-06-15

**Authors:** Particia Gužvinec, Giuseppe Muscogiuri, Maja Hrabak-Paar

**Affiliations:** 1 School of Medicine, University of Zagreb, 10000 Zagreb, Croatia; 2 ASST Papa Giovanni XXIII, 24127 Bergamo, Italy; 3 University Hospital Center Zagreb, 10000 Zagreb, Croatia

**Keywords:** computed tomography, aortopulmonary septal defect, aortopulmonary window, congenital heart disease

## Abstract

An aortopulmonary septal defect or aortopulmonary window (APW) is a rare cardiovascular anomaly with direct communication between the ascending aorta and the main pulmonary artery leading to a left-to-right shunt. It is accompanied by other cardiovascular anomalies in approximately half of patients. In order to avoid irreversible sequelae, interventional or surgical treatment should be performed as soon as possible. Cardiovascular CT, as a fast, non-invasive technique with excellent spatial resolution, has an increasing role in the evaluation of patients with APW, enabling precise and detailed planning of surgical treatment of APW and associated anomalies if present. This article aims to review the anatomical and clinical features of aortopulmonary septal defect with special emphasis on its detection and characterization by a CT examination.

## 1. Introduction

An aortopulmonary septal defect, also referred to as an aortopulmonary window (APW), is a direct side-to-side vascular shunt between the ascending aorta and the main pulmonary artery in the presence of two distinct semilunar valves [1,2]. It is one of the rarest congenital heart diseases and makes up about 0.1% to 0.6% of all cardiac heart defects [3]. It accounts for males more frequently than females in a ratio of 3:1 [4]. Due to the left-to-right shunt, it may lead to pulmonary hypertension, right heart failure, and irreversible pulmonary vascular damage. Therefore, an early and accurate diagnosis and treatment are crucial for preventing the development of these irreparable changes. In the literature, several imaging reports of patients with an aortopulmonary septal defect as an isolated anomaly or associated with other cardiovascular abnormalities can be found. This article aims to review the anatomical and clinical features of this cardiovascular anomaly with special emphasis on findings detected using cardiovascular computed tomography (CT).

## 2. Anatomy and Pathophysiology of Aortopulmonary Window

Aortopulmonary septal defect occurs if there is incomplete septation of the great arteries due to a failure of fusion of the two opposing conotruncal ridges that are responsible for separating the truncus arteriosus into the aorta and pulmonary artery. It may occur just above the semilunar valves or anywhere distally along the course of the ascending aorta and the main pulmonary artery [5]. Several classification systems of APW have been proposed, and the most commonly used Mori’s categorization divides aortopulmonary septal defect into three types: type 1 or proximal defect, type 2 or distal defect, and type 3 or total defect with a complete absence of the aortopulmonary septum [6]. Berry further divided the Mori type 2 classification into type 2A where the right pulmonary artery originates from the main pulmonary artery and type 2B where the right pulmonary artery has an anomalous origin from the aorta and is often associated with aortic arch hypoplasia and patent ductus arteriosus [7]. The Society of Thoracic Surgeons added a fourth ‘intermediate’ category which is similar to the total defect but has adequate superior and inferior rims and is most suitable for transcatheter device closure [1]. Each of these types can occur either as an isolated anomaly or accompanied by a wide range of heart defects, including ventricular septal defect (VSD), atrial septal defect (ASD), pulmonary atresia, interrupted aortic arch, etc. Berry syndrome consists of a distal aortopulmonary septal defect accompanied by hypoplasia or interruption of the aortic arch, the aortic origin of a right pulmonary artery, an intact interventricular septum, and a patent ductus arteriosus [7,8].

There are many serious complications of the aortopulmonary window. Patients develop a left-to-right shunt due to a septal opening between the systemic and pulmonary circulation. Therefore, oxygenated blood from the left ventricle flows back into the pulmonary artery in systole during early manifestation, thus increasing pulmonary blood flow and vascular resistance. Eventually, these changes, if untreated, may lead to early and rapid development of pulmonary hypertension, right heart failure, and irreversible pulmonary vascular damage [9,10].

## 3. Clinical Presentation

The clinical presentation of aortopulmonary septal defects is dependent on the size of the defect and on the associated anomalies, like VSD, ASD, interrupted aortic arch, and aortic coarctation. Patients with an aortopulmonary window have a left-to-right shunt, so clinical presentation appears similar to other left-to-right shunts, including a VSD or patent ductus arteriosus (PDA). Usually, the communication between the ascending aorta and the pulmonary artery is large and non-restrictive, resulting in congestive heart failure and pulmonary edema during the first weeks of life (usually between the second and eighth weeks of life), after the postnatal fall in pulmonary vascular resistance with progressive shunting from the systemic to the pulmonary circulation [1]. The most common symptoms and signs include diaphoresis (especially with feeding), tachypnea, tachycardia, poor weight gain, increased respiratory symptoms with viral infections, failure to thrive, and signs of congestive heart failure [11]. A clinical examination usually reveals a tachypneic infant with accessory respiratory muscle use, an enlarged heart with hyperdynamic precordium, bounding pulses, and a systolic murmur along the left sternal border, usually without a diastolic component to the murmur. Cyanosis may be present if other cyanotic anomalies (tetralogy of Fallot, transposition of the great arteries) are present, or if Eisenmenger syndrome with a shunt reversal occurs. A chest X-ray may show cardiomegaly with increased pulmonary vascular markings due to increased pulmonary blood flow [12]. In cases where the pulmonary vascular resistance does not decrease significantly after birth, patients may be asymptomatic with more subtle clinical findings. With modern imaging technology, the diagnosis is usually made prenatally or during neonatal age; rarely, the anomaly may be diagnosed later during childhood or even adulthood, most commonly in cases of smaller, restrictive defects [13].

## 4. Role of CT in the Evaluation of Patients with Congenital Heart Disease

Traditionally, first-line diagnostic methods in congenital heart disease (CHD) evaluation have been echocardiography and conventional catheter angiography, both of which have potential limitations listed above. Multidetector computed tomography (CT) can alleviate the pitfalls associated with traditional cardiac diagnostic tools. It has several advantages in CHD evaluation, including fast image acquisition and a high spatial resolution. Compared to a typical resolution of magnetic resonance imaging (MRI) of 1–2 mm, CT is a superior imaging tool in providing spatial resolution [14]. State-of-the-art CT scanners hold a notably impressive gantry rotation time below 500 milliseconds, with a minimum gantry rotation time of 230 milliseconds [15]. Consequently, CT provides a comprehensive evaluation of the anatomy of complex cardiac anomalies, furthermore, it is possible to evaluate extracardiac structures such as the aorta, systemic veins, pulmonary veins, and pulmonary arteries [16]. Additionally, multidetector CT provides a multi-planar and three-dimensional (3D) evaluation of acquired data, hence providing a better understanding of the complex anatomy of the cardiovascular system [17]. For optimal results, an electrocardiographic (ECG) gating of CT examination is required to minimize the movement of intracardiac and extracardiac structures during scanning. CT may also be useful if cardiac re-operation is planned providing information about postoperative anatomy, including complications, retrosternal distance to the right ventricular free wall, and vascular access evaluation.

The main drawbacks of CT imaging include exposure to ionizing radiation, potential hypersensitivity and nephrotoxic risks of iodinated contrast media, and limited functional information. Several strategies can provide a reduction in CT radiation doses, especially if the newest technology CT scanners are available [18]. These strategies involve the selection of predefined pediatric protocols, additional protocol optimization, and the advancement of technical parameters according to as low as reasonably achievable (ALARA) and as low as diagnostically acceptable (ALADA) principles. Techniques regarding protocol include limiting the CT scan to the area of interest and using correct ECG triggering. If retrospective ECG gating is applied, the heart is scanned during the whole cardiac cycle with a significantly higher radiation dose in comparison to prospective ECG triggering when images are acquired during systole or diastole only. The radiation dose can also be reduced by decreasing the tube voltage to 70–80 kVp, which exponentially decreases radiation dose, or by decreasing the tube current (mA), thereby increasing the pitch value, avoiding multiphase imaging, and using size-appropriate bowtie filters [19]. The noise at the low-kVp images can be reduced by iterative reconstruction algorithms. By using optimal protocols on the newest generation CT scanners, it is possible to minimize the radiation dose and reach the submilisievert dose levels [20].

The use of iodinated contrast is mandatory for the CT evaluation of cardiovascular anomalies. However, the administration of the contrast agent needs to be carefully evaluated, considering that the iodine contrast agent is only a relative rather than an absolute contraindication in patients with chronic kidney disease stages 4 and 5. Post-contrast acute kidney injury (PC-AKI) refers to a rise in serum creatinine above 0.3 mg/dL (or 26.5 µmol/L) or beyond 1.5 times the baseline level, occurring within 48–72 h after the intravenous introduction of a contrast agent. The European Society of Urogenital Radiology (ESUR) provides guidelines for renal adverse reaction prevention [21]. It stresses the importance of determining at-risk patients and calculating an estimated glomerular filtration rate (eGFR). It also offers protocols for preventive hydration. Guidelines recommend intravenous administration regimens, including 0.9% saline or 1.4% sodium bicarbonate in pre-contrast and post-contrast medium injection for patients with eGFR less than 30 mL/min/1.73 m^2^.

Additionally, although CT gives superb morphological details of the cardiac and extracardiac structures, the information about the ventricular and valvular function may be very limited, especially if prospective ECG-triggering or high-pitch scanning protocols are selected to reduce the radiation dose. Therefore, CT is supplementary to the functional information obtained by echocardiography, and in many patients, invasive diagnostic cardiac catheterization may be avoided with the optimal use of these two techniques.

## 5. CT Acquisition and Postprocessing

**Patient preparation:** Depending on the type and the speed of the available CT scanner, procedural sedation or general anesthesia may be required for younger and non-compliant patients to reduce motion-related artifacts [18]. The need for general anesthesia may be minimized by shortening the scanning time, i.e., by increasing tube rotation time and table speed (high-pitch scanning), or by using up to 16 cm wide detectors with extended coverage per rotation. Using high-pitch dual-source or wide-detector CT scanners, it is possible to scan the whole heart during a single cardiac beat minimizing the motion artifacts. To reduce the radiation dose, the scan range should be limited to the body region of interest with the extremities and external radioopaque materials (tubes, lines, and monitoring leads) positioned outside of the scanning area, and the region of interest positioned at the isocenter of the gantry. Routine administration of nitrates is not recommended before pediatric cardiac CT. The preferable site of contrast injection is the antecubital fossa, optimally on the right-sided arm to avoid streak artifacts from the left innominate vein that affect the visualization of the ascending and transverse aorta [20]. A dose of 1–2 mL/kg of iodinated contrast medium should be injected by an automated injector at a rate ranging from 1 to 5 mL/s depending on the age of the patient and the size of the intravenous cannula (optimal cannula size is 20- to 22-gauge). A biphasic contrast injecting protocol should be preferred, so the contrast injection should be followed by a saline bolus to wash out the concentrated contrast agent from the tubing and systemic veins.

**Image acquisition:** Precontrast CT scanning should be avoided since it does not add any clinically relevant information and can even double the radiation dose. The postcontrast image acquisition should be performed during the maximum opacification of vessels of interest. The optimal scanning timing after contrast injection should be determined using one of two bolus timing techniques (bolus tracking or test bolus). In APW patients, high-quality images are usually obtained if the scan starts when the contrast reaches the thoracic aorta. During the same scan, pulmonary arteries can be nicely depicted due to shunting through the aortopulmonary septal defect. Delayed scanning is usually not required for the APW diagnosis. Scanning parameters should be defined following the ALARA and ALADA principles and the above-mentioned dose reduction strategies. Furthermore, the exposure control systems should be used to automatically modulate the tube current. End-systolic acquisition (40–45% of the R-R interval) is recommended in pediatric patients who typically have fast heart rates (>75 beats per minute) [18]. Thin slices should be reconstructed to increase the spatial and isotropic resolution with a 50% increment to avoid a partial volume effect. However, very thin collimation and slice thickness may increase both the radiation exposure and the image noise [22]. The image noise should be reduced by applying iterative or novel deep learning-based reconstruction techniques whenever available [23].

**Postprocessing:** The image analysis should start by scrolling through the source thin axial images. However, the relationship between different cardiovascular structures can be much better determined using multiplanar reformatted images and 3D volume-rendered images. Additionally, the aortic diameter should be measured on a double oblique multiplanar image in the plane perpendicular to the vessel centerline [24]. Clinicians including cardiac surgeons are more familiar with multiplanar and 3D images than with axial CT images, and these reconstructions can significantly improve the communication between radiologists and clinicians. There is a growing role of 3D printing of cardiovascular models that may be used for planning CHD surgical repair enabling a thorough understanding of a patient’s anatomy [25].

## 6. CT Findings of Aortopulmonary Septal Defect

**Figure 1:**
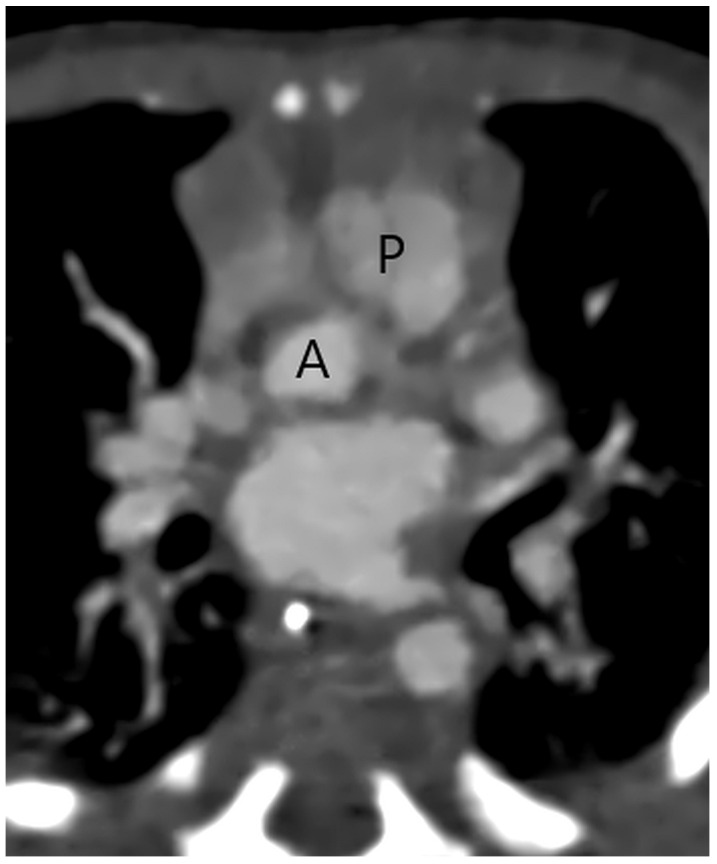
A separate aortic (A) and pulmonary (P) valve in a patient with a type 2 aortopulmonary septal defect at CT angiography.

CT with its isotropic 3D imaging properties is an excellent tool for detailed morphological evaluation of aortopulmonary septal defect and assessment of other associated cardiovascular anomalies, enabling planning of surgical or interventional treatment. Firstly, in a patient with suspect APW, it is important to identify two separate semilunar (aortic and pulmonary) valves (Figure 1) to be able to differentiate APW from a common truncus arteriosus where a single truncal semilunar valve is present.

**Figure 2:**
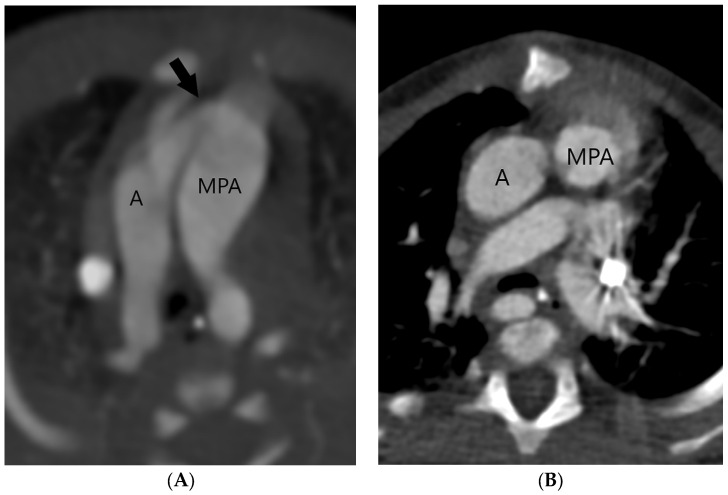
Type 1 or proximal type of aortopulmonary septal defect (arrow) is located just above the semilunar valve between the ascending aorta (A) and the right wall of the main pulmonary artery (MPA) (**A**). The right-sided aortic arch can also be detected. After surgery, the ascending aorta (A) and the main pulmonary artery (MPA) are separated (**B**).

**Figure 3:**
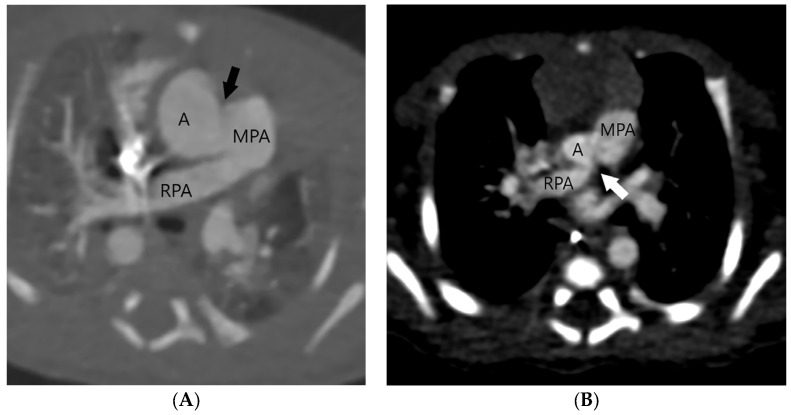
Two patients (**A**,**B**) with a type 2 or distal type of aortopulmonary septal defect (arrows) located between the distal part of the ascending aorta (A) and the anterior wall of the origin of the right pulmonary artery (RPA). Main pulmonary artery (MPA).

The aortopulmonary septal defect is located between the semilunar valves and the branch pulmonary arteries. If an APW is identified on CT, its type should be determined. Type 1 or (Figure 2) Type 2 APW is located between the distal part of the ascending aorta and the anterior wall of the origin of the right pulmonary artery (Figure 3), whereas type 3 is a total defect that involves the entire aortopulmonary septum or ascending aorta [1]. Type 4 or an intermediate type has similar features to the total defect but has adequate superior and inferior rims and is most suited for device closure [1]. Table 1 summarizes key points in the CT evaluation of patients with an aortopulmonary window.

The radiological report in a patient with aortopulmonary septal defect should define the APW type according to Mori’s categorization as one of the three types: type 1 or proximal defect, type 2 or distal defect, and type 3 or total defect [6]. It is necessary to detect two separate semilunar valves to be able to differentiate APW from the common truncus arteriosus where a single truncal semilunar valve is present. Additionally, it is important to define the exact size of the aortopulmonary septal defect, as well as the diameter of the main pulmonary artery and the ascending aorta at typical sites (aortic annulus, sinus of Valsalva, sinotubular junction, and tubular segment of the ascending aorta) [24]. Furthermore, the relationship between the aortopulmonary septal defect and the origin of coronary arteries should be defined. Besides aortopulmonary septal defect, it is also important to precisely define other cardiovascular anomalies that could be detected during the same CT examination.

## 7. APW Treatment and Prognosis

The treatment of aortopulmonary septal defect primarily involves surgical repair that should be performed as soon as possible, with transcatheter closure reserved for select cases [26]. The defect is usually closed surgically using a patch repair; however, smaller defects can be closed primarily with double ligation or suture closure [27]. The transcatheter repair using double disk closure devices is typically performed in patients with small restrictive defects in which the risk of anomalous origin of the coronary arteries is low, specifically those with a more distal APW location [5,28]. The only contraindication for APW closure is irreversible pulmonary hypertension with shunt reversal [27]. Therefore, cardiac catheterization should be utilized when the diagnosis is made after infancy to evaluate the pulmonary vascular resistance, followed by reactivity testing if there is a pulmonary vascular disease to determine whether the APW closure is still recommended [29].

Precise planning of APW closure is required, since an iatrogenic anomalous origin of the right pulmonary artery from the aorta after APW ligation has been described [30]. Other possible complications include a residual aortopulmonary septal defect, recurrent laryngeal nerve injury with hoarseness or choking during feeding, and postoperative stenosis of the ascending aorta or the main or branch pulmonary arteries [5,31]. The pulmonary artery or aortic stenosis may be treated by balloon angioplasty or stent implantation if needed. In the case of balloon angioplasty, the procedure should be cautiously performed since an AP window can theoretically recur with aggressive angioplasty. If a complication is suspected or if the evaluation of additional cardiovascular anomalies is required, a CT scan may be repeated after APW repair. The normal postsurgical finding includes a complete separation of the ascending aorta from the main pulmonary artery (Figure 2B).

Surgical repair of a simple APW is straightforward and has mortality approaching zero in most institutions if the repair occurs early in infancy [5]. Patients with APW and interrupted aortic arch also have low operative mortality with good outcomes. Mortality is almost always due to concomitant cardiovascular anomalies rather than surgical repair of the APW itself [32].

If the APW repair is not performed early enough, Eisenmenger syndrome with severe pulmonary hypertension and heart failure may develop [33]. In the case of Eisenmenger syndrome with right-to-left shunting, APW closure is contraindicated. The prognosis of patients with untreated large APW defects is very poor, with 40% mortality in the first year of life. Very rarely untreated cases may survive into adulthood.

## 8. Associated Cardiovascular Anomalies

**Figure 4:**
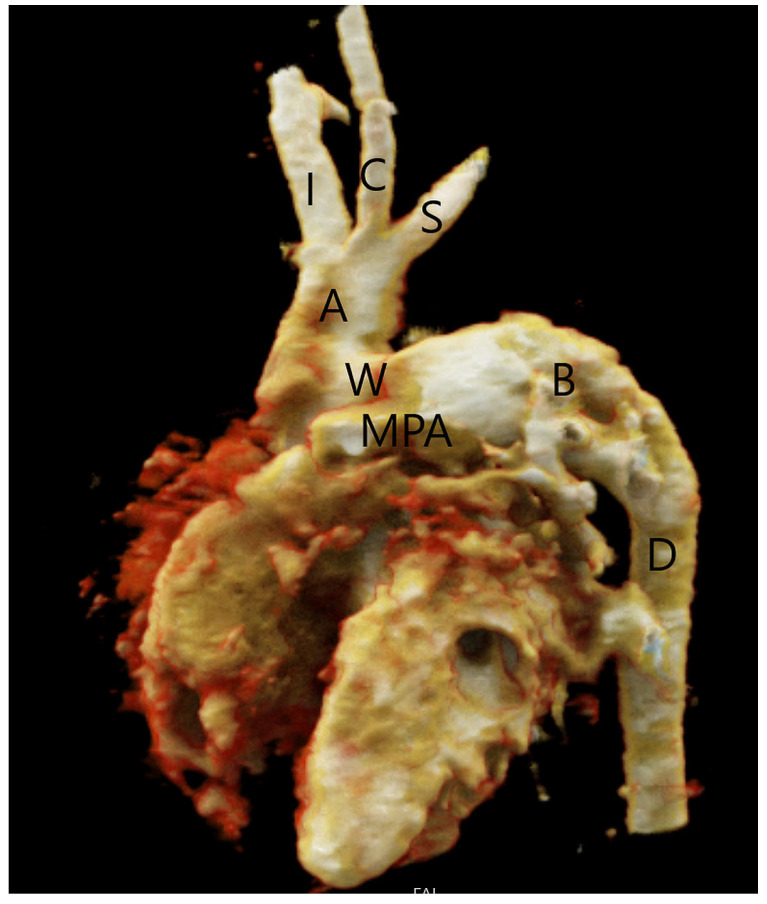
Type A interrupted aortic arch in a patient with aortopulmonary septal defect type 2 (W), volume rendering reconstruction of CT angiography. The aorta (A) ends distally to the origin of the left subclavian artery (S). The descending aorta (D) is supplied by the patent ductus arteriosus (B). Main pulmonary artery (MPA), innominate artery (I), and left common carotid artery (C).

The aortopulmonary window presents as either an isolated anomaly or in association with other anomalies. Around 50% of the patients have associated cardiovascular anomalies requiring surgical repair, of which the most common are type A interrupted aortic arch distal to the left subclavian artery (Figure 4) and aortic coarctation that are present in 10–15% of patients with aortopulmonary septal defect [5]. In these patients, flow to the caudal part of the body is preserved through the aortopulmonary window and then via the patent ductus arteriosus to the descending aorta. Therefore, these patients require prostaglandin therapy to maintain ductal patency and blood supply of the caudal part of the body. In patients with associated aortic arch abnormalities, a cardiogenic shock may occur if the ductus arteriosus closes. Other associated anomalies described in the literature include tetralogy of Fallot, the right aortic arch, transposition of the great arteries, ASD, VSD, and an abnormal origin of the coronary arteries that may arise from the edge of the defect on the aortic or pulmonary artery side of the defect [5,34]. For that reason, the imperative is to correctly assess the anatomical relationship between the aorta and pulmonary artery, as well as those of the heart, great vessels, and aortic arch.

**Figure 5:**
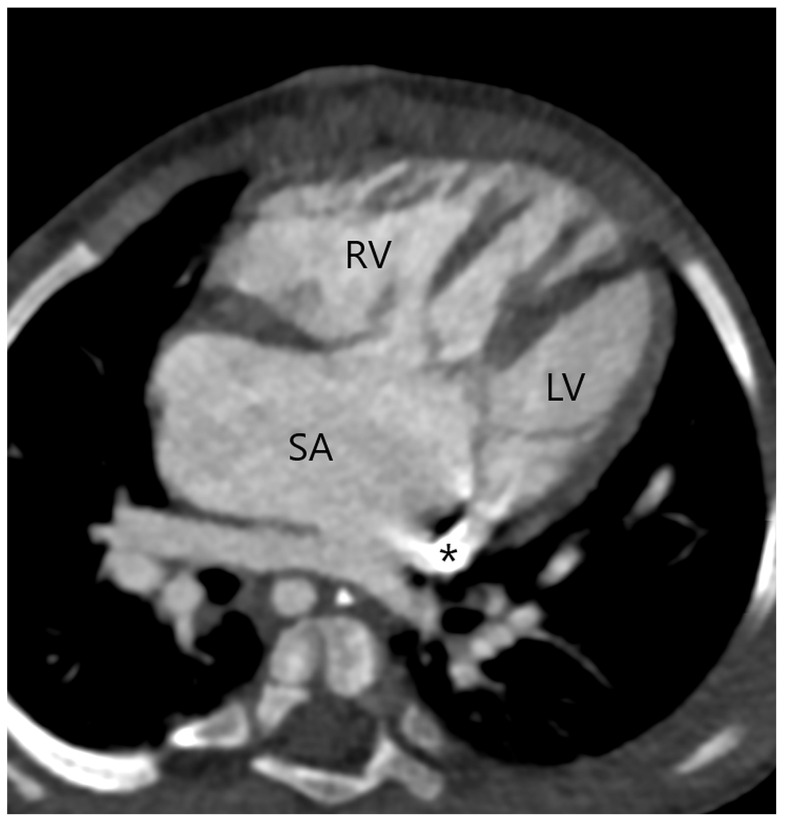
A single atrium (SA) with a complete atrioventricular septal defect in a patient with a type 1 APW. A persistent left superior vena cava is draining into the coronary sinus (asterisk). Right ventricle (RV); left ventricle (LV).

**Figure 6:**
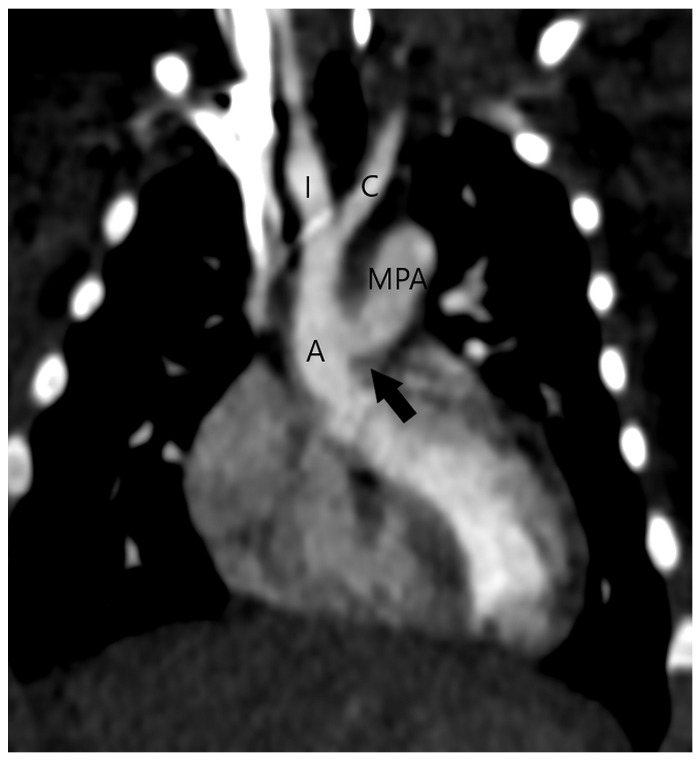
Type B interrupted aortic arch in a patient with aortopulmonary septal defect type 2 (arrow); multiplanar reconstruction of CT angiography. The aorta (A) ends with bifurcation on the innominate artery (brachiocephalic trunk, I) and the left common carotid artery (C). Main pulmonary artery (MPA).

It is possible to overlook the aortopulmonary window when several heart defects are present. CT has proven to be accurate in imaging the aortopulmonary window in association with other congenital heart defects, even in patients showing inconclusive echocardiographic results. One of our patients with a type 1 APW had a single atrium with a complete atrioventricular septal defect (Figure 5) that was corrected during a later surgical procedure. Although patients with APW nearly always have a type A interrupted aortic arch, our patient with aortopulmonary septal defect type 2 had a type B interrupted aortic arch (Figure 6), but without DiGiorge syndrome that is frequently associated with a type B interrupted aortic arch. In a previous report, CT efficiently demonstrated an aortopulmonary window associated with type A interrupted aortic arch, ASD, VSD, and PDA [35]. In another case, an echocardiogram that suggested only a VSD was followed by a CT examination that identified type 2 APW and excluded additional cardiac abnormalities [36]. In a patient where an echocardiogram suggested truncus arteriosus and VSD, CT successfully revealed a type 1 APW and a perimembranous VSD suggestive of tetralogy of Fallot with membranous pulmonary atresia [37]. Moreover, in patients with pulmonary atresia and ventricular septal defect, an APW may be the sole source of pulmonary blood flow in the absence of PDA and major aortopulmonary collateral arteries [30]. A congenital unilateral absence of the right pulmonary artery with a VSD and a wide aortopulmonary window has also been described [38].

## 9. Discussion

Multimodality cardiovascular imaging is crucial for early recognition and timely APW treatment. There is an increasing role of noninvasive multimodality imaging (echocardiography, MRI, and CT) in the evaluation of CHD patients, with a decreasing number of diagnostic invasive catheter angiography procedures that should be reserved for situations when non-invasive methods cannot establish the correct diagnosis and to uncover associated heart defects. Furthermore, if the APW diagnosis is made after infancy, catheter angiography is mandatory for pulmonary vascular resistance measurement and vasoreactivity testing to assess whether APW closure is recommended [10]. The main disadvantages of this technique are the high radiation dose, its invasive nature, and iodine contrast administration during the catheterization procedure. In selected patients, catheter angiography may be used for transcatheter APW closure.

Transthoracic echocardiography is a first-line, non-invasive, widely used diagnostic method. Multiple studies demonstrate the significant value of echocardiography for diagnosing APW, both prenatally and postnatally [39,40,41]. It is a widely available, portable, non-ionizing tool that provides immediate results. Echocardiography allows direct visualization of the defect of the wall between the aorta and pulmonary artery from different views, including parasternal short and long-axis views, high parasternal short-axis views, and subcostal coronal views. At Doppler echocardiography, an aortopulmonary communication is confirmed by an abnormal continuous forward flow in the pulmonary arteries. Unlike patients with a PDA, APW patients have a retrograde flow in the transverse arch during diastole. Despite the advantages of echocardiography, the APW cannot always be visualized since the method has certain limitations, such as an operator-dependence and a narrow field of view for assessing extracardiac vascular structures, such as pulmonary arteries and veins, the aorta, and cavopulmonary shunts, especially in patients with poor echocardiographic windows. Also, the aorta-to-pulmonary artery pressure difference can be diminished or even reversed by the early development of severe pulmonary arterial hypertension, decreasing or inverting blood flow through the defect, respectively [40]. As a result, Doppler features may not be conclusive [42]. Similarly, a simple APW may be missed by fetal echocardiography because of the equal pressure in the fetal ascending aorta and pulmonary artery, resulting in minimal detectable flow through the defect [1].

Magnetic resonance imaging has become more widely used in recent years. The strengths of this imaging tool include a lack of radiation exposure and a detailed evaluation of cardiac anatomy and blood flow dynamics. In contrast, the main limitations of this method include long acquisition time, contraindications related to ferromagnetic foreign bodies and implants, claustrophobia-related patient discomfort, and eventually administration to gadolinium-based contrast agent [43]. Additionally, in children younger than 7 years and uncooperative patients, general anesthesia is required to avoid motion artifacts. However, cardiovascular MRI should be the modality of choice for a comprehensive morphological and functional assessment of cooperative CHD patients after the age of 7 years.

Multidetector or dual-source CT examination is a fast, available, and safe non-invasive imaging modality that gives excellent and very detailed morphological information about the intracardiac and extracardiac structures including the coronary arteries. Therefore, CT is an outstanding imaging technique for additional morphological assessment after the echocardiography in patients with aortopulmonary septal defects detected during infancy and early childhood.

## 10. Conclusions

An aortopulmonary septal defect is usually closed surgically with a good prognosis if it is simple and corrected early enough. Cardiovascular CT, as an available and fast imaging technique with superb spatial resolution, is an excellent noninvasive imaging technique for planning APW surgery and analysis of additional cardiovascular anomalies.

## 11. Future Directions

Multimodality non-invasive imaging modalities including cardiovascular CT enable a precise definition of different cardiovascular anomalies. Therefore, invasive catheter angiography becomes unnecessary in many clinical scenarios and should be reserved for the evaluation of patients with pulmonary hypertension and those undergoing interventional transcatheter treatment. Three-dimensional-printed models created from a CT examination may be helpful in surgical planning and simulation of cardiac surgery procedures, thus further improving patient management [44].

## Figures and Tables

**Table 1:** Key points in the CT evaluation of patients with aortopulmonary window.

Two separate semilunar (aortic and pulmonary) valves. Direct side-to-side communication between the ascending aorta and the main pulmonary artery anywhere between the semilunar valves and the branch pulmonary arteries. Four types: Proximal type, just above the sinus of Valsalva and a few millimeters above the semilunar valve; Distal type, between the distal part of the ascending aorta and the anterior wall of the origin of the right pulmonary artery; Total defect involves the entire aortopulmonary septum; Intermediate type, similar to the total defect but has adequate superior and inferior rims for device closure Common additional anomalies: Type A interrupted aortic; Aortic coarctation; Tetralogy of Fallot; Right aortic arch; Transposition of the great arteries; ASD; VSD; Abnormal origin of the coronary arteries.

## Data Availability

No new data were created.
